# Molecular Epidemiology of G6PD Genotypes in Different Ethnic Groups Residing in Saharan and Sahelian Zones of Mauritania

**DOI:** 10.3390/pathogens10080931

**Published:** 2021-07-23

**Authors:** Oum Kelthoum Mamadou Djigo, Mohamed Salem Ould Ahmedou Salem, Sileye Mamadou Diallo, Mohamed Abdallahi Bollahi, Boushab Mohamed Boushab, Aymeric Garre, Nasserdine Papa Mze, Leonardo Basco, Sébastien Briolant, Ali Ould Mohamed Salem Boukhary

**Affiliations:** 1 Unité de Recherche “Génomes et Milieux” (Jeune Equipe Associée à l’Institut de Recherche pour le Développement), Faculté des Sciences et Techniques, Université de Nouakchott Al-Aasriya, Nouakchott, Mauritania; mari1702@live.fr (O.K.M.D.); salem0606@yahoo.fr (M.S.O.A.S.); diallosiley08@gmail.com (S.M.D.); 2 Institut National de Recherche en Santé Publique (INRSP), Nouakchott, Mauritania; bollahi@yahoo.com; 3 Department of Internal Medicine and Infectious Diseases, Kiffa Regional Hospital, Assaba, Mauritania; bboushab@gmail.com; 4 Aix Marseille Université, Institut de Recherche pour le Développement (IRD), Assistance Publique-Hôpitaux de Marseille (AP-HM), Service de Santé des Armées (SSA), Vecteurs—Infections Tropicales et Méditerranéennes (VITROME), 13005 Marseille, France; aymericgarre@gmail.com (A.G.); npapamze@gmail.com (N.P.M.); lkbasco@yahoo.fr (L.B.); sbriolant@wanadoo.fr (S.B.); 5 Institut Hospitalo-Universitaire (IHU)—Méditerranée Infection, 13005 Marseille, France; 6 Unité de Parasitologie Entomologie, Département de Microbiologie et Maladies Infectieuses, Institut de Recherche Biomédicale des Armées (IRBA), 13005 Marseille, France

**Keywords:** DNA sequencing, ethnic groups, epidemiology, G6PD deficiency, malaria elimination, Mauritania, PCR-RFLP, *Plasmodium vivax*

## Abstract

*Plasmodium vivax* malaria is endemic in Mauritania. Individuals with glucose-6-phosphate dehydrogenase (G6PD) deficiency may develop acute hemolytic anemia when exposed to 8-aminoquinoline antimalarial drugs, which are indispensable for a complete cure. The prevalence of *G6PD* allelic variants was assessed in different ethno-linguistic groups present in Mauritania. A total of 996 blood samples (447 males and 549 females; 499 white Moors and 497 individuals of black African ancestry) were collected from febrile patients in 6 different study sites: Aleg, Atar, Kiffa, Kobeni, Nouakchott, and Rosso. The presence of the African-type *G6PD* A- (G202A, A376G, A542T, G680T, and T968C mutations) and the Mediterranean-type *G6PD* B- (C563T) variants was assessed by PCR followed by restriction fragment length polymorphism and/or DNA sequencing. The prevalence of African-type *G6PD* A- genotype was 3.6% (36/996), with 6.3% (28/447) of hemizygote (A-) males and 1.5% (8/549) of homozygous (A-A-) females. Forty of 549 (7.3%) women were heterozygous (AA-). The following genotypes were observed among hemizygous men and/or homozygous women: A376G/G202A (22/996; 2.2%), A376G/T968C Betica-Selma (12/996; 1.2%), and A376G/A542T Santamaria (2/996; 0.2%). The Mediterranean-type *G6PD* B- genotype was not observed. The prevalence rates of *G6PD* A- genotype in male (10/243; 4.1%) and heterozygous female (6/256; 2.3%) white Moors were lower (*p* < 0.05) than those of males (18/204; 8.8%) and heterozygous females (34/293; 11.6%) of black African ancestry. There were only a few homozygous women among both white Moors (3/256; 1.2%) and those of black African ancestry (5/293; 1.7%). The prevalence of G6PD deficiency in Mauritania was comparable to that of neighboring countries in the Maghreb. Because of the purportedly close ethnic ties between the Mauritanian white Moors and the peoples in the Maghreb, further investigations on the possible existence of the Mediterranean-type allele are required. Moreover, a surveillance system of G6PD phenotype and/or genotype screening is warranted to establish and monitor a population-based prevalence of G6PD deficiency.

## 1. Introduction

Glucose-6-phosphate dehydrogenase (G6PD) is the key enzyme of pentose phosphate pathway, the unique source of the reduced form of nicotinamide adenine dinucleotide phosphate (NADPH) in mature human erythrocytes. NADPH plays a key role in enzyme-based antioxidant system to protect erythrocytes from oxidative damage. When mature erythrocytes are deficient in G6PD and are exposed to various exogenous trigger factors, including infections, a variety of drugs (e.g., 8-aminoquinoline antimalarial drugs, which include primaquine and tafenoquine), and vicine and convicine found in fava beans, the antioxidant defense system is unable to neutralize free radicals that accumulate in the erythrocytes, leading to lipid peroxidation and cell lysis, which may be manifested clinically by acute hemolytic anemia [1,2].

In humans, G6PD deficiency is an X-linked recessive genetic trait which occurs frequently, affecting approximately 400 million people worldwide [3,4,5,6]. The *G6PD* gene consists of 13 exons and 12 introns encoding a protein with a mass of 59 kDa [7]. The gene is highly polymorphic with more than 200 mutations reported worldwide [8,9]. Despite the high number of mutant *G6PD* variants, only a few of them result in a functionally deficient G6PD enzyme. On the African continent, the most prevalent G6PD variants of clinical importance are the African-type G6PD A- found in sub-Saharan Africa and the Mediterranean-type G6PD B- variant mainly found in North Africa. Most *G6PD* mutations are clinically silent unless the G6PD-deficient individual is challenged with a trigger factor. The main clinical manifestation in such individuals is potentially fatal non-immune acute hemolytic anemia.

G6PD deficiency is a major issue in areas where *Plasmodium vivax* malaria is present because 8-aminoquinolines are the only currently available drugs that can kill hypnozoites, the latent liver forms of *P. vivax* (and also *P. ovale*). The hypnozoites remain dormant for weeks or months after the primoinfection and are reactivated due to still unknown mechanisms, leading to new malarial attacks called relapse [10,11]. Although most acute attacks due to *P. vivax* are not fatal, each attack may worsen anemia. The therapeutic objective in *P. vivax*-infected patients is complete elimination of both blood and liver stage parasites [12]. However, primaquine and tafenoquine given at the standard doses for anti-hypnozoite therapy are contraindicated in G6PD-deficient patients. These patients are therefore subject to multiple *P. vivax* (or *P. ovale*) malaria attacks from a primoinfection and can also become a source of further transmission to other persons, rendering the objective of malaria elimination difficult to attain.

The endemicity of *P. vivax* malaria has been firmly established over the past two decades in the Saharan zone of Mauritania, including Nouakchott, the capital, and Atar, an oasis city [13,14,15,16]. Faced with this new reality, since 2013, the Mauritanian health authorities recommend the administration of primaquine, in addition to either artesunate-amodiaquine or artemether-lumefantrine, to prevent relapse in *P. vivax*-infected patients with normal G6PD activity [17]. However, this treatment regimen has not yet been deployed in health facilities due to the lack of point-of-care G6PD testing prior to primaquine administration.

At present, the amount of data available on *G6PD* genotypes (or phenotypes) in Mauritania is limited to two studies conducted in Nouakchott [18,19]. It is known that *G6PD* polymorphisms are associated with ethnicity [8,20]. Mauritania is home to different ethnolinguistic groups distributed into two large groups, the Moorish group comprising the white Moors (also locally called Bidhanes or Beydanes) and the black Moors (also called Haratin) and the Black African group made up of the Pular, Soninke and Wolof [21]. There are no census-based official numbers of people belonging to each ethnic group because the Mauritanian government no longer publishes the proportions of ethnic group since the 1977 census.

We hypothesized that some white Moors, who are of North African Arab-Berber descent, may have inherited the Mediterranean-type *G6PD* B- genotype [22,23]. All other ethnic groups are of black African ancestry. Based on this information, as well as on earlier studies carried out in sub-Saharan African countries bordering Mauritania and in Mauritania, it was further hypothesized that some individuals belonging to the ethnic groups of black African descent may carry the African-type *G6PD* A- genotype [4,18,19,24,25,26,27].

Given the scarcity of data on *G6PD* genotypes in the general Mauritanian population, the present study was conducted to determine the prevalence of African-type and Mediterranean-type *G6PD* genotypes in both men and women of all ethnic groups present in Mauritania and establish a baseline genetic database as the first step to the full implementation of primaquine therapy in *P. vivax*-infected Mauritanian patients in the near future.

## 2. Results

### 2.1. Characteristics of Participants

A total of 996 blood samples (447 men and 549 women) were analyzed for *G6PD* polymorphisms. The following numbers of samples collected from 6 study sites were genotyped: 101 from Atar, 226 from Nouakchott, 79 from Aleg, 20 from Kiffa, 323 from Kobeni, and 247 from Rosso (Table 1 and Table 2). Based on ethnic groups, 499 (50.1%) blood spots were from white Moors, 316 (31.7%) from black Moors, 134 (13.5%) from Pular, 24 (2.4%) from Wolof, and 23 (2.3%) from Soninke.

### 2.2. Prevalence and Type of G6PD Genotype and Gender

Overall, 419 of 447 (93.7%, 95% confidence interval (95% CI), 91.1–95.8%) men had normal *G6PD* genotypes A (*n* = 52) or B (*n* = 367), and 28 (6.3%, CI95% (4.2–8.9%)) were A- (Table 1). Among women, 501 of 549 (91.3%, 95% CI (88.6–93.5%)) carried the normal *G6PD* genotypes AA, BB or AB, whereas 40 (7.3%, 95% CI (5.3–9.8%)) were heterozygous (AA- or BA-), and 8 (1.5%, 95% CI (0.6–2.9%)) were African-type homozygous A-A- (Table 2). The global prevalence of African-type genotypes probably associated with G6PD deficiency therefore consisted of 6.3% (28/447, 95% CI (4.2–8.9%)) hemizygous males and 1.5% (8/549, 95% CI (0.6–2.9%)) homozygous females, i.e., a total of 3.6% (36/996, 95% CI (2.5–5.0%)) in both sexes.

Among the individuals with *G6PD* A- genotypes, *G6PD* A^-(202)^ or A376G/G202A double mutation was observed in 2.2% (22/996, 95% CI (1.4–3.3%); 19 hemizygous males and 3 homozygous females) (Table 3 and Table 4). The second most frequently observed African-type *G6PD* genotype associated with G6PD deficiency was Betica-Selma or A376G/T968C double mutation (12/996; 1.2%, 95% CI (0.6–2.1%); 7 males and 5 homozygous females). Santamaria *G6PD* A- or A376G/A542T was observed in 2 males, i.e., with a frequency of 0.2% (2/996, 95% CI (0–0.7%)); there was no homozygous Mauritanian females with this African-type *G6PD* genotype. Among heterozygous females, the most common genotype was 376A/G-202G/A (*n* = 23/549 females, 4.2%, 95% CI (2.7–6.2%)), followed by 376A/G-T968T/C (*n* = 15/549 females, 2.7%, 95% CI (1.5–4.5%)) and 376A/G-542A/T (*n* = 2/549 females, 0.4%, 95% CI (0–1.3%)). We did not find any individuals carrying the A376G/G680T African-type *G6PD* genotype. Likewise, none of the patients included in the present study had the Mediterranean-type *G6PD* B- genotype.

Data are expressed as the number of individual males belonging to one of the ethnic groups (*n*) and percentage in each locality. *G6PD* genotype: African-type genotype in hemizygous male, A-; African-type normal male with a single A376G mutation, A; normal male, B. There were no male Pulars in Kiffa and no male Wolofs in Kobeni.

Data are expressed as the number of individual females belonging to one of the ethnic groups (*n*) and percentage in each locality. *G6PD* genotype: African-type genotype in homozygous female, A-A-; African-type genotype in female heterozygous carrier, AA- or BA-; normal female with a single African-type A376G mutation with either AA or BA and normal female without any mutation, BB. There were no female Black Moors in Atar, no female Wolofs in Aleg, and no female Soninkes in Rosso in the present study.

### 2.3. Prevalence and Type of G6PD Genotype and Ethnic Groups

Table 3 and Table 4 summarize the data on individuals with African-type *G6PD* genotype in relation to ethnic groups. Among white Moors with African-type *G6PD* variant (19/499, 3.8%, 95% CI (2.3–5.9%)), 10 males were hemizygous, 3 females were homozygous, and 6 females were heterozygous. Among black Moors with African-type *G6PD* variant (39/316, 12.3%, 95% CI (8.9–16.5%)), 15 males were hemizygous, 3 females were homozygous, and 21 females were heterozygous. Among the individuals belonging to the minority ethnic groups composed of Pulars, Wolofs, and Soninkes (19/181, 10.5%, 95% CI (6.4–15.9%)), 3 males were hemizygous, 2 females were homozygous, and 13 females were heterozygous. Taken together, there were no statistically significant difference between the number of males of black African ancestry (18/204; 8.8%, 95% CI (5.3–13.6%)) and the male white Moors (10/243; 4.1%, 95% CI (2.0–7.4%)),) who carried African-type *G6PD* genotype associated with G6PD deficiency (*p* = 0.0772). Likewise, there were significantly more heterozygous females among those of black African origin (34/293; 11.6%, 95% CI (7.9–15.3%)) than heterozygous white Moor women (6/256; 2.3%, 95% CI (0.9–5.0%)) (*p* < 0.0001). There was no statistically significant difference between the number of homozygous females of black African ancestry (5/293; 1.7%, 95% CI (0.6–3.9%)) and that of homozygous female white Moors (3/256; 1.2%, 95% CI (0.2–3.4%)) (*p* = 0.73), but the numbers of homozygous females were too low for a meaningful statistical comparison.

Data expressed as the number (*n*) of affected males with African-type genotype among the total number (N) of males belonging to each ethnic group and percentage of affected males in each ethnic group in parentheses. The proportion of A- allelic variants refers to the type of A- variants in relation to 28 males (i.e., denominator) with the African-type *G6PD* variant. There were a total of 447 men in the study.

Data expressed as the number (*n*) of affected females with African-type genotype among the total number (N) of females belonging to each ethnic group and percentage of affected females in each ethnic group in parentheses. The proportion of A- allelic variants refers to the type of A- variants in relation to 48 heterozygous or homozygous females (i.e., denominator) with the African-type *G6PD* variant. There were a total of 549 females in the study. Heterozygous 680G/T and homozygous 542T/T and 680T/T genotypes were not observed.

### 2.4. Distribution of G6PD Genotypes in Mauritania

**Figure 1:**
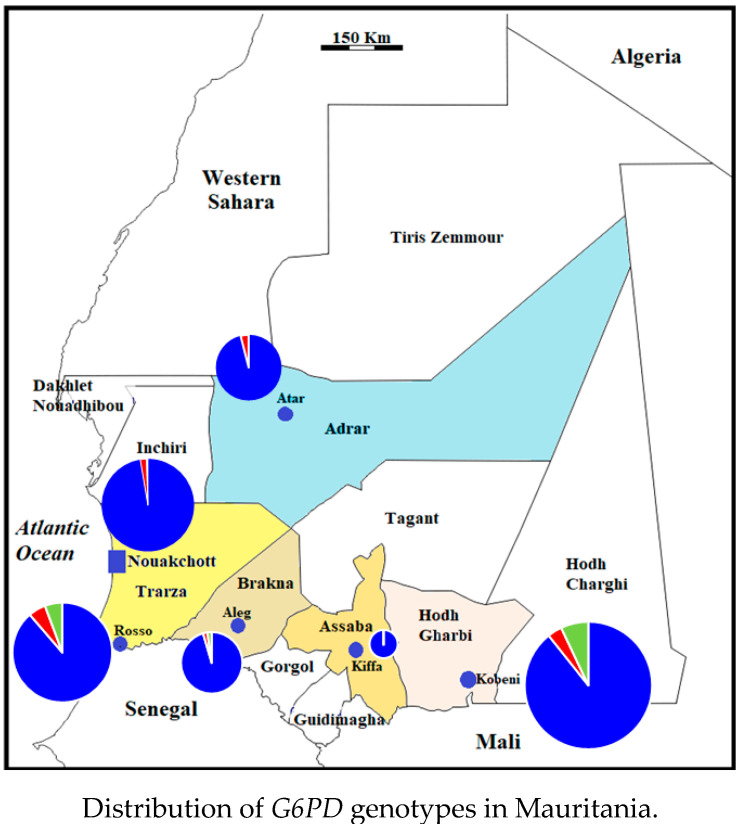
The proportions of *G6PD* normal genotypes, genotypes associated with G6PD deficiency, and heterozygous females in six study sites in Mauritania are presented. Blue (AA, BB, and AB normal females and A and B normal hemizygous males); red, A- hemizygous males and A-A- homozygous females; green, AA- and BA- heterozygous females.

The distribution of *G6PD* genotypes in six study sites in Mauritania is depicted in Figure 1. Hemizygous or homozygous genotypes associated with the African-type G6PD deficiency were identified in all six study sites, except for Kiffa. However, the situation in Kiffa was not representative of the general population in the city due to the small number of samples available from this study site. If data from Kiffa are excluded, the proportions of hemizygous and homozygous genotypes associated with G6PD deficiency ranged from 2.2% (in Aleg and Nouakchott) to 5.7% (in Rosso). Heterozygous females tended to be more present in southern Sahelian regions of the country where relatively high numbers of Mauritanians of black African ancestry reside, as compared to the desertic regions in the north.

## 3. Discussion

One of the hypotheses of the present study that some white Moors may carry the Mediterranean-type G6PD deficiency seems to be invalidated. The results of the present study are in contradiction with those of our earlier study based on multiplex allele-specific PCR [18]. In that study, of 44 phenotypically G6PD deficient samples according to the rapid diagnostic test of G6PD, 13 (8 white Moors, 3 black Moors, and 2 black Africans) were reported to have the Mediterranean variant. These 13 samples were re-analyzed by the same multiplex allele-specific PCR kit, PCR-RFLP protocol described in the present study, and DNA sequencing, and they were all characterized to be of normal *G6PD* genotype by three methods (data not shown). We can therefore tentatively conclude that, based on more than 500 samples (499 from the present study + 8 from the earlier study) from white Moors in six different study sites in Mauritania, the Mediterranean-type G6PD B- variant was not found in our study.

It can be hypothesized that Mediterranean-type *G6PD* genotype is rare (< 0.2%, i.e., less than 1 in 500 based on the present study) or possibly absent in white Moors despite the supposed ethnic and historical ties with peoples from North Africa. It has been estimated that the prevalence rates of G6PD deficiency in the general population in Algeria and Morocco, two neighboring Maghreb countries north of Mauritania, are 2.8% and 0.6%, respectively [28]. In Algeria, an early study had shown that 3.2% of males living in Algiers, the capital city, have G6PD deficiency [29]. A later study conducted in Algerian males with clinical manifestations associated with G6PD deficiency (favism, neonatal jaundice, acute hemolytic anemia) demonstrated that 23% (23/100) were due to the Mediterranean-type *G6PD* genotype, while 46% had the African-type A- genotype [22]. In a study performed in Morocco, 15% (54/360) of anemic patients were found to be G6PD-deficient, of whom 50% were characterized as the African-type G6PD A- and 7.4% had the Mediterranean-type variant [30]. By contrast, our study showed that all genotypes associated with G6PD deficiency were of African type among Mauritanians. Another hypothesis that may explain the absence of the Mediterranean-type G6PD deficiency in Mauritanians in the present study is the possibility that white Moors may have had in reality less ties with peoples in North Africa and remained geographically isolated from them by the Sahara Desert for centuries. The white Moors are composed of several social strata (warriors or “hassani,” religious leaders or “zawaya,” vassals or “zenaga,” artisans, and bards or “griots”). Some of these social strata are known to have intermarried with black African populations over the past centuries [21], which could have diluted the prevalence of G6PD B- disorder, if ever it was present to begin with. These hypotheses on the genetic relatedness of Mauritanian white Moors and peoples dwelling in North Africa today could be further strengthened by performing genetic analysis of other codons that have been reported to undergo unique mutations in some Algerian and Moroccan subjects with G6PD deficiency, such as the Kabyle and Aures G6PD A- variants [22,23,29]. These considerations need further historical and comparative human genome analysis in order to fully understand why Mediterranean-type *G6PD* genotype was not found in Mauritanians in our study.

The data presented here support the other hypothesis of this study that some Mauritanians of black African origin may be affected by the African-type G6PD deficiency. Among men belonging to black African ancestry (i.e., black Moors and one of three minority ethnic groups), 18 men (18/204; 8.8%, 95% CI (5.3–13.6%)) had the A- genotype. This prevalence rate in hemizygous Mauritanian men of black African origin is comparable to that reported for Senegalese males belonging to Sereer ethnic group (10%) and Malian males belonging to the Bambara ethnic group (10.5%), but higher than that of Dogon (2.2%) and Malinké (4.0%) ethnic groups in Mali [25,31,32].

Although we found a much higher prevalence of G6PD deficiency in black Moors and three minority ethnic groups than in white Moors in Mauritania, in practice, it may be argued that many of these individuals of black African ancestry may not be confronted with the potential problem of anti-hypnozoite therapy with 8-aminoquinolines because they tend to be Duffy-negative, as in the majority of black Africans elsewhere in sub-Saharan Africa, and may be naturally “resistant” to *P. vivax* infections [33,34,35,36]. However, cases of *P. vivax* infections have been reported in recent years in Duffy-negative individuals in Africa, including in Mauritania [35,36,37]. At present, *P. vivax* infection in Duffy-negative individuals is generally thought to be more of an exception rather than the rule, and the underlying biochemical and molecular mechanisms are poorly understood [38,39]. Furthermore, many of the minority ethnic groups of black African ancestry reside in southern Mauritania, where the chances of being exposed to the risk of *P. vivax* malaria are slim [40,41,42,43,44,45]. Nonetheless, many Mauritanians living in the south often travel to the north, where *P. vivax* is endemic, and some migrate to the northern Saharan zone, often for economic reasons, especially to Nouakchott, which has become a melting pot of various ethnic groups. Mauritanians of all ethnic backgrounds are therefore exposed to the risk of *P. vivax* infection, albeit to a lesser extent in those with black African ancestry due to the relative protection provided by the absence of Duffy antigen on the surface of red blood cells which serves as the principal known receptor for entry of *P. vivax* merozoites into the cell [36].

G6PD A^-^ is generally associated with mild to moderate deficiency (class III, i.e., 10–60% residual activity, except for Santamaria, class II, i.e., less than 10% residual activity) and G6PD B^-^ with severe deficiency (class II) [8,20,46]. Based on the WHO classification, the data obtained in the present study suggest that Mauritanians with mutant *G6PD* genotype are likely to present phenotypically mild to moderate deficiency. However, this assumption is not supported by solid evidence, and WHO experts have recommended against the use of this old classification to make clinical decisions [47]. The results of the present study are in agreement with earlier studies conducted in other West African countries which showed that, among different mutations resulting in African-type G6PD deficiency, *G6PD*^−202^ is most frequently observed [25]. In several previous studies, A542T, G680T, and T968C mutations were not observed in West Africa (Burkina Faso, Ghana, Mali, and Nigeria) [25,27]. In Mauritania, A376G/A542T Santamaria and A376G/T968C Betica-Selma were found, but not A376G/G680T.

Regardless of the A- variant and the ethnic group to which an individual *P. vivax*-infected patient belongs, G6PD screening test or standard biochemical test is mandatory before treatment with a standard 14-day regimen of primaquine can be considered. If the person is G6PD normal male, the standard dose of primaquine can be given if a qualitative or quantitative G6PD test displays normal enzymatic activity (> 60% of normal). If the person is G6PD normal female, the standard dose of primaquine can be given only if a reliable quantitative G6PD test is available and confirms a normal G6PD activity [12]. If a Mauritanian individual is G6PD-deficient, it is likely to be due to the mutant African-type *G6PD* genotype. The clinician would have to decide on a case-by-case basis whether a modified scheme of anti-hypnozoite therapy with primaquine (once weekly dose for 8 weeks) would be beneficial and outweigh possible risks [12]. In some cases, as in heterozygous females and even G6PD normal females with normal qualitative G6PD screening test but without quantitative test confirmation, a precise measurement of G6PD enzyme activity is required before making a decision.

It has been estimated that the worldwide prevalence of G6PD deficiency is 5% and that the prevalence is highest in Africa (8.5%) [3,4,5,6]. In the present study, the prevalence of G6PD deficiency based on genotyping was 6.3% in males and 1.5% in homozygous females, or 3.6% if the prevalence of both genders is combined. Data obtained in the present work, in particular samples from Kobeni city, were slightly skewed towards ethnic groups of black African ancestry and were not representative of the proportions of different ethnic groups present in Kobeni. Moreover, the samples used in the present study were collected from febrile patients in health centers and hospitals, where persons with an adequate financial resource and access to health tend to visit more often. Data obtained in health structures do not reflect the situation in the general population. A population-based study will be required for a more accurate assessment of the distribution of *G6PD* genotypes in the country. In addition, in some study sites, especially in the oasis city of Atar, none of the black African ethnic groups were seen at the health center. These considerations probably led to sampling bias in our study, with white Moors (50.1%, including men and women) over-representing the general population. The actual proportions of the ethnic groups of black African ancestry in Mauritania today are not known. Nevertheless, their proportion (18.2% composed of 13.5% Pular, 2.4% Wolof, and 2.3% Soninke) observed in the present study remains most likely less than what is expected. These confounding factors may have considerably modified the representativity of different ethnic groups in the samples, which may, at least in part, explain the lower-than-expected prevalence of African-type G6PD deficiency observed in the present study. This limitation of the study is counterbalanced by the fact that samples were collected from febrile patients spontaneously presenting themselves to health centers and hospitals. A subset of these patients may have been infected by *P. vivax* and may have required primaquine therapy.

Our study has other limitations. The present study was focused on the well-known *G6PD* 202, 376, 542, 680, and 968 polymorphisms associated with African-type G6PD A^-^ and *G6PD* 563 associated with the Mediterranean-type G6PD B^-^. Other less common mutations were not analyzed. Although one of our initial hypotheses about the possible presence of the Mediterranean variant associated with moderate G6PD deficiency among white Moors turned out to be unconfirmed among the study participants, preconceived ideas should not preclude investigators and clinicians to rule out G6PD deficiency. An unexpected variant with severe phenotypic deficiency may sometimes be detected in Africa [48]. Moreover, due to the lack of sophisticated equipment and pre-existing technical know-how, G6PD phenotype was not characterized using the gold standard method, which is spectrophotometry. A study correlating *G6PD* genotype and G6PD phenotype is being planned to address this gap in the current knowledge of G6PD epidemiology in the country.

## 4. Materials and Methods

### 4.1. Study Sites

**Figure 2:**
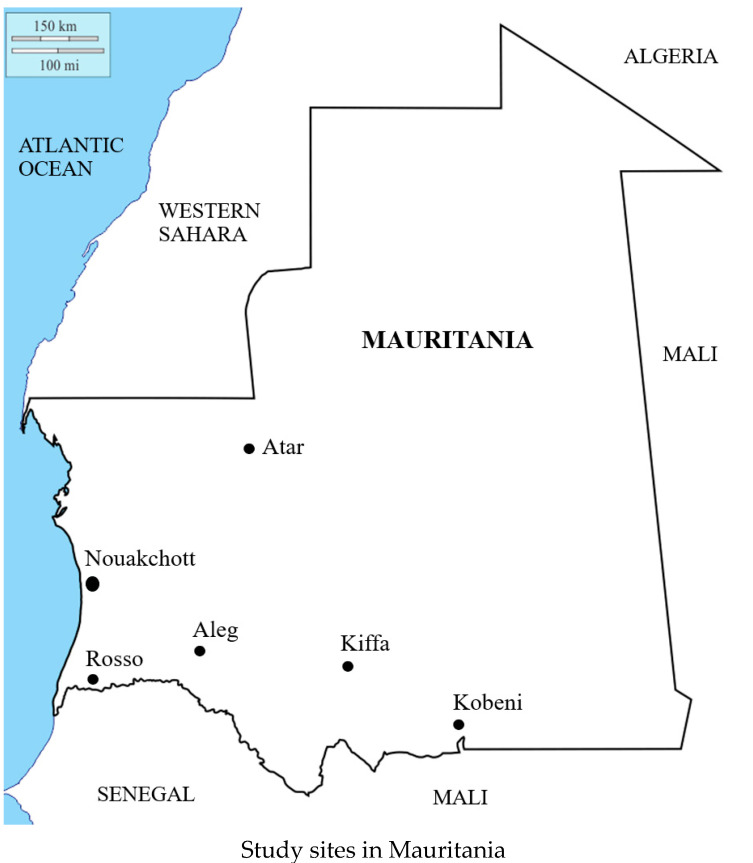
Study sites in Mauritania. Nouakchott and Atar are situated in the Saharan zone. Kobeni, Kiffa, and Aleg are in the Sahelo-Saharan transition zone. Rosso is located in the Sahelian zone along the Senegal River.

Field studies were conducted in 2015–2018 in the following cities in Mauritania, from the north to the south, located in six of 12 provinces (locally called “Wilaya”) of Mauritania (Figure 2): Atar (20°31′00” N 13°03′00” W; 38,877 inhabitants, according to the latest available data from the National Statistic Office [49], the regional capital of Adrar, located in northern Saharan region; Nouakchott (18°06′ N 15°57′ W; 958,399 inhabitants), the capital city of Mauritania where one-fourth of the population reside; Aleg (17°3′ N 13°55′ W; 111,512 inhabitants), the regional capital of Brakna in central Mauritania; Kiffa (16°37′ N 11°24′ W; 110,714 inhabitants), the regional capital of Assaba, southern Mauritania, located about 600 km from Nouakchott; Kobeni city (15°49′02” N 9°24′39” W; 92,690 inhabitants) in the province of Hodh Elgharbi, southeastern region of the country near the Mauritania-Malian border; and Rosso (16°30′46” N 15°48′18” W; 57,726 inhabitants), the regional capital of Trarza, located along the Senegal River in southwestern Mauritania along the border between Mauritania and Senegal. In total, approximately 63% of the total Mauritanian population reside in these cities [49].

Blood samples were collected in the following public health structures: health center of Atar; Teyarett health center and Maternity and Infant Hospital Center (Centre Hospitalier Mère et Enfant) in Nouakchott; health center of Aleg; Department of Internal Medicine and Infectious Diseases, Kiffa Regional Hospital, Kiffa; health center of Kobeni; and health center of Rosso. Except for Kiffa and Aleg (which is located midway between Nouakchott and Kiffa), these study sites were selected on the basis of previous series of field surveys carried out by our research teams to assess malaria epidemiology in three different settings defined by geoclimatic conditions, namely, the northern Saharan zone (Atar and Nouakchott), southern and southeastern Sahelo-Saharan transition zone (Kiffa and Kobeni; also Aleg), and southwestern Sahelian zone (Rosso) [13,16,40,41,42,45,50]. In addition, Atar, Nouakchott, Rosso, and Kobeni are sentinel sites of the Mauritanian National Malaria Control Programme [51,52].

### 4.2. Collection of Blood Samples

Fingerprick capillary blood samples were collected as part of the series of epidemiological studies conducted in the country between 2015 and 2018 [15,16,45,50]. Patients were recruited if they were febrile or had a recent history of fever within 48 h prior to consultation, regardless of age or sex. After explaining the purpose of the study and obtaining an informed consent, two to three drops (100–150 µL) of fingerprick capillary blood were imbibed onto Whatman grade 3MM filter paper (GE Healthcare UK Ltd., Little Chalfont, Buckinghamshire, UK) or Whatman FTA card (GE Healthcare), air dried, and stored in a sealed plastic sachet with a desiccant at −20 °C until DNA extraction.

### 4.3. Genotyping

A 1 mm-diameter disc of filter paper imbibed with dried blood was punched out and placed in 96-well plates. Human DNA was extracted using an automated MagMAX™-Express system (Thermo Fisher Scientific, Montigny-le-Bretonneux, France) according to the manufacturer’s instructions. At the end of the DNA extraction protocol, approximately 120 µL of supernatant containing DNA were obtained from each sample.

**Figure 3:**
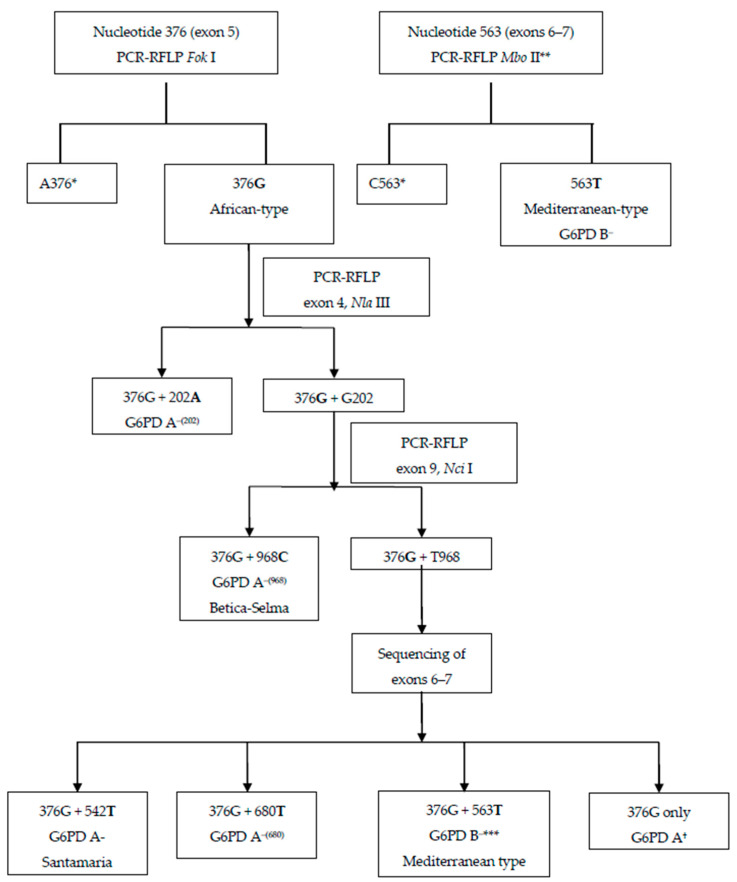
Flow diagram depicting PCR-sequencing strategy to analyze *G6PD* genotypes. * If a sample was characterized as wild-type in nucleotides 376 and 563, it was considered as wild-type *G6PD* genotype in this study. ** The PCR-amplified fragment of exons 6-7 was sequenced in samples with A376G mutation if digestion did not display G202A or T968C mutation. *** For samples with A376G, but without G202A or T968C mutation, sequencing further confirmed the result of PCR-RFLP. ^†^ African-type G6PD A denotes the presence of A376G mutation, without a second mutation in nucleotide 202, 542, 680, or 968. The single mutation in 376 does not change the G6PD phenotype (i.e., the individual has a normal G6PD activity).

In the present study, DNA fragments including the following *G6PD* nucleotide positions, where mutations have been reported to occur most frequently in Africa, giving rise to G6PD A- variant, were amplified by PCR and analyzed by restriction fragment length polymorphism (RFLP) and/or DNA sequencing in a stepwise manner: 376, 202, 542, 680, and 968 [25] (Figure 3). In addition, nucleotide 563 was characterized in all samples to determine the presence of C563T mutation, which gives rise to the Mediterranean variant (G6PD B-).

Genomic human DNA was first amplified using 2.5–5 µl of eluted DNA template and primer pairs A376G_F (5′-CCCAGGCCACCCCAGAGGAGA-3′) and A376G_R (5′-CGGCCCCGGACACGCTCATAG-3′; 0.5 µM each) in a PCR master mix containing DreamTaq DNA polymerase, buffer, 2 mM MgCl_2_, and deoxyribose nucleoside triphosphate (dNTP) (Thermo Fisher DreamTaq™ Green PCR Master mix; ThermoFisher Scientific, Illkirch, France) and the following thermal cycling program: initial step of 95 °C for 5 min, 35 cycles of 95 °C for 1 min, 53 °C (annealing temperature) for 1 min, and 72 °C for 1 min, then an extension at 72 °C for 10 min. PCR products (308 base pairs (bp)) were incubated at 37 °C overnight (at least 18 h) with *Fok* I (also called *Bse*G I or *Bts*C I) endonuclease (ThermoFisher Scientific, Illkirch, France).

PCR amplification was also performed for all samples to determine nucleotide 563 using the primer pairs C563T_F (5′-ACTCCCCGAAGAGGGGTTCAAGG-3′) and C563T_R (5′-CCAGCCTCCCAGGAGAGAGGAAG-3′) (0.5 µM each) [53]. The PCR program was as follows: initial step of 95 °C for 5 min, 35 cycles of 95 °C for 1 min, 63 °C (annealing temperature) for 1 min, and 72 °C for 1 min, then an extension at 72 °C for 10 min. PCR products (547 bp) were incubated at 37 °C overnight (at least 18 h) with *Mbo* II endonuclease (ThermoFisher Scientific, Illkirch, France).

Samples with A376G nucleotide substitution were further analyzed to detect the presence of an additional mutation in 202, 542, 680, or 968. The 216-bp fragment spanning nucleotide 202 was amplified using the primer pairs G202A_F (5′-CCACCACTGCCCCTGTGACCT-3′) and G202A_R (5′-GGCCCTGACACCACCCACCTT-3′) in DreamTaq™ Green PCR Master mix [25]. The thermal cycler was programmed as follows: initial denaturation at 95 °C for 5 min, 35 cycles of 95 °C for 1 min, 65 °C (annealing temperature) for 1 min, and 72 °C for 1 min, and an extension at 72 °C for 10 min. The PCR product was incubated at 37 °C overnight with *Nla* III (also called *Hin* 1 II) (ThermoFisher Scientific, Illkirch, France).

If nucleotide 202 was wild-type, PCR was performed to amplify a 283-bp fragment spanning nucleotide 968 using the primer pairs T968C_F (5′-TCCCTGCACCCCAACTCAAC-3′) and T968C_R (5′-CCAGTTCTGCCTTGCTGGGC-3′) [25]. PCR program was as for other fragments, with the anneal temperature of 65 °C. The amplicons were digested by incubating at 37 °C overnight with *Nci* I (ThermoFisher Scientific, Illkirch, France). After enzymatic digestion, digested amplificons were analyzed by 2% agarose gel electrophoresis. The PCR conditions and primers are summarized in Table 5.

The 547-bp fragment spanning nucleotides 542, 563, and 680, which was initially amplified for RFLP using *Mbo* II to determine the nucleotide 563 in all samples, was sequenced if samples with A376G mutation had neither G202A nor T968C mutation. Sequencing was outsourced (Macrogen Europe; Amsterdam, The Netherlands).

F for forward primer; R for reverse primer; Tm, annealing temperature. The size in base pairs (bp) refers to the expected fragment size of the PCR product. Three restriction sites were analyzed in the fragment spanning exons 6 and 7. The primer sequences of the fragments amplified from exons 4, 5, and 9 (G202A, A376G, T968C) were published by Carter et al. [25], and those in exons 6 and 7 (A542T, C563T, G680T) were published by Ezz El-Deen et al. [53]. The published primer sequence of C563T_F has one missing C. This was corrected based on the complete human *G6PD* gene sequence (GenBank accession number X55448.1) [54].

### 4.4. Data Analysis

In all study sites, samples were randomly selected for analysis, with the exception of patients enrolled in Kobeni where, due to a large number of available samples (more than 2300) [45], 327 samples were selected based on the following arbitrary criteria: absence of malaria parasites and preference for patients belonging to one of the minority groups of black African origin. These latter groups are therefore over-represented in Kobeni in the present study.

The wild-type phenotype of normal enzyme is designated G6PD B. The corresponding genotype in hemizygous male is “B” for haplotype GAACGT (based on nucleotides G202/A376/A542/C563/G680/T968) and “BB” for homozygous normal female. The African variant genotype with a single A376G nucleotide change is associated with the phenotype G6PD A, which is not associated with any change in enzyme activity compared to the normal enzyme. The corresponding genotypes are denoted “A” in hemizygous male (haplotype G**G**ACGT) and AA or AB in female. The genotypes denoted A and B in males and AA, AB, and BB in females are considered G6PD normal. The African-type deficient enzyme is designated G6PD A-; there are two mutations, one of which is A376G. The corresponding genotypes are “A-” in hemizygous male (haplotypes **AG**ACGT, G**GT**CGT, G**G**AC**T**T, or G**G**ACG**C**) and “A-A-” in homozygous deficient female. The genotypes of heterozygous females are either “AA-” or “BA-.” The Mediterranean variant with C563T (haplotype GAA**T**GT) is designated Mediterranean G6PD B^-^.

If A376G nucleotide change (AAT to GAT; amino acid substitution Asn126Asp, exon 5) occurs, the genotype is either *G6PD* A or *G6PD* A^-^. *Fok* I yields two bands (116 bp + 192 bp) in the presence of the mutant 376G allele, but it has no restriction site in the PCR fragment with the wild-type A376 allele (a single 308-bp band). A heterozygote female with *G6PD* A or A^-^ genotype is characterized by the presence of three bands (116 bp + 192 bp + 308 bp).

Enzymatic digestion of the fragment spanning nucleotide 202 with *Nla* III yields two fragments (81 bp + 135 bp) if it carries the mutant allele 202A, one uncut 216-bp fragment if it has the wild-type allele G202, or three fragments (81 bp + 135 bp + 216 bp) in heterozygous females. The genotype with double mutations A376G and G202A is designated “*G6PD* A^−(202)^.”

*Nci* I restriction enzyme digests the 283-bp fragment into two (121 bp + 162 bp) fragments in the presence of mutation T968C or into three fragments (121 bp + 162 bp + 283 bp) in heterozygous females. The genotype characterized by double mutations A376G and T968C is called “*G6PD* A^-^ Betica-Selma.”

Sequencing of some PCR products of samples with A376G mutation, but without G202A or T968C, allowed not only to determine A542T and G680T mutations but also to confirm PCR-RFLP results using *Mbo* II to determine nucleotide 563. The genotypes with double mutations A376G and A542T or A376G and G680T are called “*G6PD* A^-^ Santamaria” and “*G6PD* A^−(680)^,” respectively [8].

*Mbo* II enzyme digests the 547-bp fragment into 4 fragments (25 + 26 + 199 + 377 bp) if there is a wild-type C563 allele and 5 fragments (25 + 26 + 100 + 199 + 277 bp) in the presence of mutant 563T (Mediterranean-type *G6PD* B-).

In the present paper, the term “normal *G6PD* genotype” is synonymous with the wild-type *G6PD* B and denotes the absence of mutation in one of the following nucleotide positions: 202, 376, 542, 563, 680, and/or 968.

Proportions were compared using a two-tailed Fisher’s exact test. The *p*-value of < 0.05 was used to test for significance.

## 5. Conclusions

This is the first nationwide survey of *G6PD* genotyping that addresses the relation between ethnic origin and G6PD deficiency in Mauritania. The African-type *G6PD* mutations were found in all ethnic groups, more frequently in those of black African ancestry than in white Moors. From a practical viewpoint, every *P. vivax*-infected Mauritanian patient would require a reliable G6PD screening test and, for females, a reliable quantitative G6PD measurement, before a combination of blood schizonticidal and anti-hypnozoite antimalarial drugs can be administered to ensure that radical cure is achieved to completely eliminate the parasites from the patient. A phenotypic and/or genotypic surveillance of G6PD deficiency is warranted as the country attempts to move on to implement anti-hypnozoite therapy as part of the malaria elimination program.

## Figures and Tables

**Table 1:** Frequency of G6PD*A and B genotypes in males in six localities in Mauritania.

Locality	Ethnic Group	N	Genotype		
			A-	A	B
Atar	White Moors	52	4 (7.7)	2 (3.8)	46 (88.5)
	Black Moors	2	0	0	2 (100)
Nouakchott	White Moors	71	1 (1.4)	2 (2.8)	68 (95.8)
	Black Moors	19	1 (5.3)	0	18 (94.7)
	Pular	9	0	1 (11.1)	8 (88.9)
	Wolof	2	0	0	2 (100)
	Soninke	1	0	0	1 (100)
Aleg	White Moors	29	0	0	29 (100)
	Black Moors	12	0	2 (16.7)	10 (83.3)
	Pular	1	0	0	1 (100)
	Wolof	1	1 (100)	0	0
Kiffa	White Moors	9	0	2 (22.2)	7 (77.8)
	Black Moors	2	0	1 (50.0)	1 (50.0)
Kobeni	White Moors	41	2 (4.9)	6 (14.6)	33 (80.5)
	Black Moors	58	7 (12.1)	12 (20.7)	39 (67.2)
	Pular	21	0	7 (33.3)	14 (66.7)
	Soninke	4	0	1 (25.0)	3 (75.0)
Rosso	White Moors	41	3 (7.3)	3 (7.3)	35 (85.4)
	Black Moors	47	7 (14.9)	11 (23.4)	29 (61.7)
	Pular	19	2 (10.5)	2 (10.5)	15 (79.0)
	Wolof	5	0	0	5 (100)
	Soninke	1	0	0	1 (100)
Total		447	28 (6.3)	52 (11.6)	367 (82.1)

**Table 2:** Frequency of G6PD*A and B genotypes in females in six localities in Mauritania.

Locality	Ethnic Group	N	Genotype					
			A-A-	AA-	BA-	AA	BB	BA
Atar	White Moors	47	0	0	0	1 (2.1)	44 (93.6)	2 (4.3)
Nouakchott	White Moors	83	1 (1.2)	0	0	1 (1.2)	70 (84.3)	11 (13.3)
	Black Moors	31	1 (3.2)	1 (3.2)	0	1 (3.2)	21 (67.8)	7 (22.6)
	Pular	5	1 (20.0)	0	0	0	4 (80.0)	0
	Wolof	3	0	0	0	1 (33.3)	2 (66.7)	0
	Soninke	2	0	0	0	0	2 (100)	0
Aleg	White Moors	23	1 (4.3)	0	0	0	21 (91.4)	1 (4.3)
	Black Moors	11	0	0	1 9.1)	2 (18.2)	6 (54.5)	2 (18.2)
	Pular	2	0	0	1 (50.0)	0	1 (50.0)	0
Kiffa	White Moors	5	0	0	0	0	5 (100)	0
	Black Moors	3	0	0	0	0	1 (33.3)	2 (66.7)
	Pular	1	0	0	0	0	0	1 (100)
Kobeni	White Moors	57	1 (1.8)	1 (1.8)	3 (5.2)	1 (1.8)	48 (84.2)	3 (5.2)
	Black Moors	77	1 (1.3)	4 (5.2)	10 (13.0)	6 (7.8)	38 (49.3)	18 (23.4)
	Pular	48	0	2 (4.2)	2 (4.2)	0	31 (64.6)	13 (27.0)
	Wolof	2	0	0	0	0	2 (100)	0
	Soninke	15	0	1 (6.7)	0	1 (6.7)	10 (66.6)	3 (20.0)
Rosso	White Moors	41	0	0	2 (4.9)	2 (4.9)	29 (70.7)	8 (19.5)
	Black Moors	54	1 (1.9)	1 (1.9)	4 (7.4)	4 (7.4)	30 (55.5)	14 (25.9)
	Pular	28	0	1 (3.6)	5 (17.9)	2 (7.1)	16 (57.1)	4 (14.3)
	Wolof	11	1 (9.1)	0	1 (9.1)	2 (18.2)	4 (36.4)	3 (27.2)
Total		549	8 (1.5)	11 (2.0)	29 (5.3)	24 (4.4)	385 (70.1)	92 (16.7)

**Table 3:** Frequency of G6PD*A- allelic variants in males with G6PD*A- genotypes in Mauritania.

Ethnic Group	*n*/N (%)	G6PD*A- Allelic Variants (*n*; % Among Men with African-Type *G6PD* Genotypes)			
		202A	542T	680T	968C
White Moors	10/243 (4.1)	6 (21.4)	2 (7.1)	0	2 (7.1)
Black Moors	15/140 (10.7)	12 (42.8)	0	0	3 (10.7)
Pular	2/50 (4.0)	0	0	0	2 (7.1)
Wolof	1/8 (12.5)	1 (3.6)	0	0	0
Soninke	0/6 (0)	0	0	0	0
Total	28/447 (6.3)	19	2	0	7

**Table 4:** Frequency of G6PD*A- allelic variants in females with G6PD*A- genotypes in Mauritania.

Ethnic Group	*n*/N (%)	G6PD*A- Allelic Variants (*n*; % Among Females with the African-Type *G6PD* Genotypes)				
		Heterozygote			Homozygote	
		202G/A	542A/T	968T/C	202A/A	968C/C
White Moors	9/256 (3.5)	5 (10.4)	1 (2.1)	0	2 (4.2)	1 (2.1)
Black Moors	24/176 (13.6)	14 (29.2)	1 (2.1)	6 (12.5)	1 (2.1)	2 (4.2)
Pular	12/84 (14.3)	3 (6.2)	0	8 (16.7)	0	1 (2.1)
Wolof	2/16 (12.5)	1 (2.1)	0	0	0	1 (2.1)
Soninke	1/17 (5.9)	0	0	1 (2.1)	0	0
Total	48/549 (8.7)	23 (47.9)	2 (4.2)	15 (31.2)	3 (6.2)	5 (10.4)

**Table 5:** PCR primers used to amplify fragments of *G6PD* gene.

Primers	Primer Sequence	Tm (°C)	Size (bp)	Enzyme
**Exon 5**				
A376G_F	5′-CCCAGGCCACCCCAGAGGAGA-3′	58	308	*Fok* I
A376G_R	5′-CGGCCCCGGACACGCTCATAG-3′			
**Exons 6-7**				
C563T_F	5′-ACTCCCCGAAGAGGGGTTCAAGG-3′	62	547	*Mbo* II (C563T)
C563T_R	5′-CCAGCCTCCCAGGAGAGAGGAAG-3′			*Acc* III (G542T) *Bst* NI (G680T)
**Exon 4**				
G202A_F	5′-CCACCACTGCCCCTGTGACCT-3′	65	216	*Nla* III
G202A_R	5′-GGCCCTGACACCACCCACCTT-3			
**Exon 9**				
T968C_F	5′-TCCCTGCACCCCAACTCAAC-3′	65	283	*Nci* I
T968C_R	5′-CCAGTTCTGCCTTGCTGGGC-3′			

F for forward primer; R for reverse primer; Tm, annealing temperature. The size in base pairs (bp) refers to the expected fragment size of the PCR product. Three restriction sites were analyzed in the fragment spanning exons 6 and 7. The primer sequences of the fragments amplified from exons 4, 5, and 9 (G202A, A376G, T968C) were published by Carter et al. [25], and those in exons 6 and 7 (A542T, C563T, G680T) were published by Ezz El-Deen et al. [53]. The published primer sequence of C563T_F has one missing C. This was corrected based on the complete human *G6PD* gene sequence (GenBank accession number X55448.1) [54].

## Data Availability

The authors confirm that the data supporting the findings of this study are available within the article.
